# Non-invasive *in vivo* study of morphology and mechanical properties of the median nerve

**DOI:** 10.3389/fbioe.2024.1329960

**Published:** 2024-04-11

**Authors:** Ruixia Xu, Lei Ren, Xiao Zhang, Zhihui Qian, Jianan Wu, Jing Liu, Ying Li, Luquan Ren

**Affiliations:** ^1^ Key Laboratory of Bionic Engineering, Ministry of Education, Jilin University, Changchun, China; ^2^ School of Medical Informatics and Engineering, Xuzhou Medical University, Xuzhou, China; ^3^ Editorial Department of Journal of Bionic Engineering, Jilin University, Changchun, China

**Keywords:** median nerve, morphology, biomechanics, shear wave elastography, ultrasound

## Abstract

The current literature studied the median nerve (MN) at specific locations during joint motions. As only a few particular parts of the nerve are depicted, the relevant information available is limited. This experiment investigated the morphological and biomechanical properties of the MN. The effects of the shoulder and wrist motions on MN were explored as well. Eight young healthy female individuals were tested with two-dimensional ultrasound and shear wave elastography (SWE). The morphological and biomechanical properties were examined in limb position 1, with the wrist at the neutral position, the elbow extended at 180°, and the shoulder abducted at 60°. In addition, the experiment assessed the differences among the wrist, forearm, elbow, and upper arm with Friedman’s test and Bonferroni *post hoc* analysis. Two groups of limb positions were designed to explore the effects of shoulder movements (shoulder abducted at 90° and 120°) and wrist movements (wrist extended at 45° and flexed at 45°) on the thickness and Young’s modulus. Differences among the distributions of five limb positions were tested as well. The ICC_3, 1_ values for thickness and Young’s modulus were 0.976 and 0.996, respectively. There were differences among the MN thicknesses of four arm locations in limb position 1, while Young’s modulus was higher at the elbow and wrist than at the forearm and upper arm. Compared to limb position 1, only limb position 4 had an effect on MN thickness at the wrist. Both shoulder and wrist motions affected MN Young’s modulus, and the stiffness variations at typical locations all showed a downward trend proximally in all. The distributions of MN thickness and Young’s modulus showed fold line patterns but differed at the wrist and the pronator teres. The MN in the wrist is more susceptible to limb positions, and Young’s modulus is sensitive to nerve changes and is more promising for the early diagnosis of neuropathy.

## 1 Introduction

As a peripheral nerve, the median nerve (MN) is formed by the medial and lateral cords of the brachial plexus (Dang and Rodner, 2009; Arnold and Elsheikh, 2013). The MN is a typically mixed nerve. The sensory axons of the MN are provided mostly from the lateral cord, while the bulk of the motor input is contributed from the medial cord (Tsai and Steinberg, 2008). It travels through the upper arm along with the brachial artery, without branches to muscles above the elbow. As it crosses the elbow and enters the forearm, the MN supplies the pronator teres, flexor carpi radials, palmaris longus, and flexor digitorum superficialis muscles (Miller and Reinus, 2010; Arnold and Elsheikh, 2013). It controls the internal rotation of the forearm, flexion and extension of the index finger and the wrist, the most thumb motions, and feedback on sensation for the radial palm.

Throughout the arm, the MN passes through many narrow structures where it may compress the nerve (Abdalbary, Abdel-Wahed et al., 2021). The carpal tunnel is the most common location for MN entrapment (Knutsen and Calfee, 2013; Kantarci, Ustabasioglu et al., 2014). In addition, other structures, such as pronator teres and biceps aponeurosis, have also been reported (Strohl and Zelouf, 2017; Balaban and Torun, 2022). Nerve compression is a common nerve injury, leading to abnormal sensation and dysfunction in the innervation area (Keir and Rempel, 2005; Neal and Fields, 2010; Gao, Weng et al., 2013). Symptoms of MN compression include weakness or paralysis of the innervated muscles, pain and sensory abnormalities, and limited muscle function. Overuse may aggravate these symptoms (Miller and Reinus, 2010), so early diagnosis and prevention of MN entrapment are particularly crucial.

Currently, nerve injury is diagnosed primarily through clinical symptoms, electrophysiological examination, and magnetic resonance imaging (MRI) (Zhu, Yan et al., 2018; Wessel, Kerluku et al., 2022). However, due to MN’s elaborate anatomical structure and its complex alignment, visually and effectively displaying a MN is a challenge for clinical and imaging research. Because of real-time dynamic visualization and flexible operation, ultrasound is widely used in neural research (Hobson-Webb, 2020; Yoshii, Zhao et al., 2020). Ultrasound studies have diagnosed neuropathy predicted on echogenic intensity and the cross-sectional area (CSA) (Klauser, Halpern et al., 2009; Aiman, Bosnjak et al., 2014; Nam, Peterson et al., 2019; Yoshii, Zhao et al., 2020; Wessel, Kerluku et al., 2022). However, high-frequency ultrasound imaging can only provide qualitative analysis. The quantitative measurement relies on the operator’s subjective judgment, leading to discrepancies and inconsistencies in detection criteria (Beekman and Visser, 2003; Aseem, Williams et al., 2017; Kluge, Langer et al., 2022).

Another characteristic of the nerve is mechanical properties, which can indirectly assess modifications in the nerve (Greening and Dilley, 2017). Nerve stiffness is a quantitative index used to study changes in nerve compression and nerve adaptation to different limb positions. However, changes in the morphology and mechanical properties of MN during adaptation to limb positions have not been comprehensively reported.

Bihui Zhu et al. investigated the effect of wrist extension on MN Young’s modulus (Zhu, Yan et al., 2018). Chelsea L. Rugel et al. studied the impacts of elbow extension on the mechanical properties of distal and proximal MN by shear wave elastography (SWE) (Rugel, Franz et al., 2020). Another study from Jane Greening demonstrated a significant increase in MN stiffness with maximal extension posture (wrist and elbow extension) (Greening and Dilley, 2017). These works offer specific theoretical support for interpreting exercise-induced neuropathy. However, findings focusing on a few typical locations in healthy individuals provide limited information (Andrade, Nordez et al., 2016). Therefore, studying the overall morphological and mechanical properties of MN could provide more comprehensive results.

In this work, we used two-dimensional ultrasound and SWE to quantitatively investigate the effects of shoulder movements and wrist motions on MN morphological and mechanical properties in a healthy population by measuring the overall distributions of thickness and Young’s modulus. The conclusions can provide reliable theoretical support for the pathophysiology of neuropathy, early diagnosis, and postoperative rehabilitation.

## 2 Materials and methods

### 2.1 Subject population

Eight healthy volunteers were enrolled in this experiment. The research method was approved by the Ethics Committee of Bethune First Hospital of Jilin University and followed the Helsinki Declaration. The main selection criteria for healthy volunteers were (1) age between 21 and 27, (2) female individuals, and (3) BMI between 18 and 25; and exclusion criteria were (1) pregnancy, (2) diseases that cause MN abnormalities such as systemic psychiatric disorders, neurological entrapment syndrome, musculo-skeletal disorders, and (3) a mechanical injury to the MN, or a history of local inflammation, trauma, tumor, or surgery in the area examined. The participants’ demographics are shown in Table 1. Every volunteer was informed of and agreed to the test procedures before the test was administered.

**TABLE 1:** Participants’ characteristics.

	Gender	Number	Age	BMI	Clinical characteristic
Subjects	Female	Eight	23 ± 1.87	18.42 ± 3.05	Healthy

### 2.2 Equipment and procedure

The device used in the experiment was the Aixplorer ultrasound system (SuperSonic Imaging, Aix-en-Provence, France) with a 2–10-MHz linear array probe.

**FIGURE 1:**
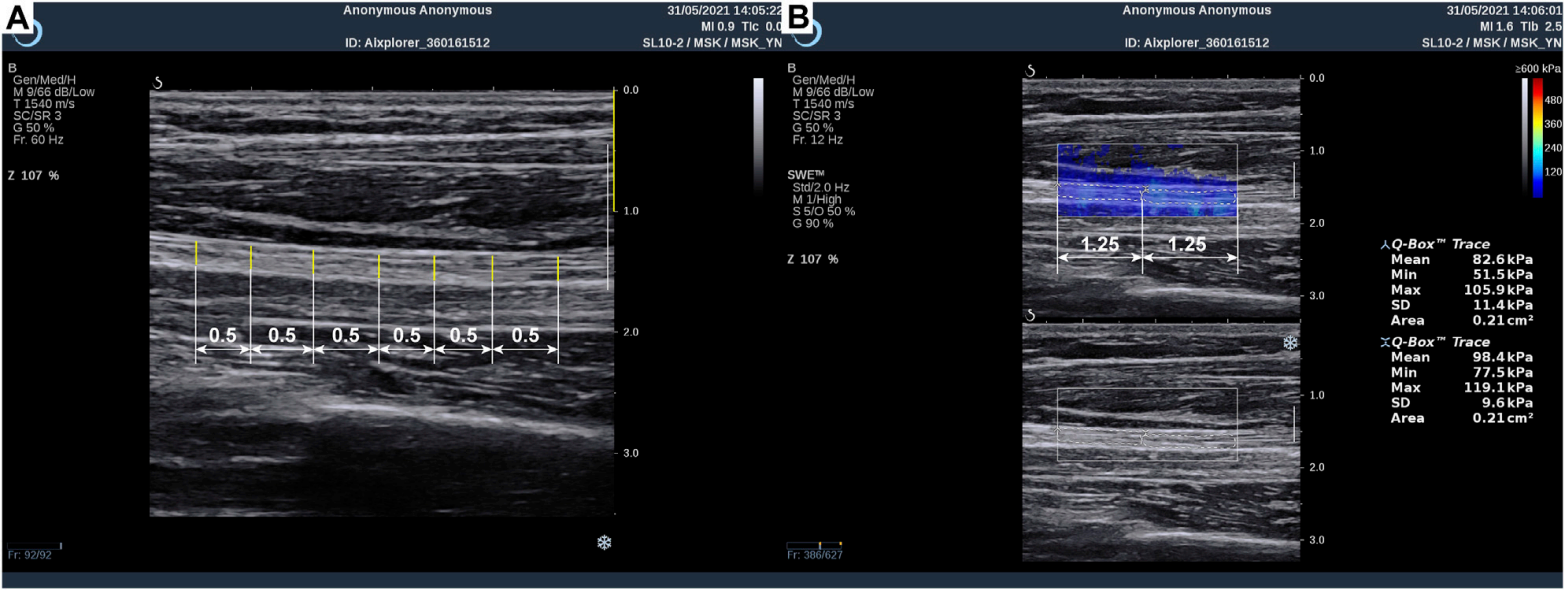
Method of median nerve ultrasonic measurement: **(A)** thickness measurement and **(B)** Young’s modulus measurement.

In each subject, the MN tested area covered the entire area from the distal wrist crease to 10 cm above the elbow, which is near the axilla. As shown in Figure 1A, the operator measured MN thickness in B-mode images at 0.5-cm intervals using Digimizer (v.5.4.1, MedCalc software, Belgium), and the images were acquired from the Aixplorer ultrasound system. First, at the starting point of the scan, the MN appeared at the upper edge of the imaging area, where the radius appeared in the lower left corner, and three clear figures were saved in dynamic imaging for measurement of nerve thickness afterward. Subsequently, Young’s modulus was measured in the SWE mode with the musculoskeletal preset of the Aixplorer. The square elastography window (region of interest, ROI) was set to 2.5 cm in width and 1 cm in height and put in the middle of the image. The built-in measurement tool Q-Box^TM^ trace was applied to outline the boundary of the MN and calculate Young’s modulus of the MN within the trace, including the maximum, average, and minimum values. The average Young’s modulus was taken into analysis. Each ROI area was divided into two equal length sections to measure the elasticity values of two different MN sites (Figure 1B).

Later, the probe was moved half the length of the probe to measure the characteristics of the MN at the next site along the extension direction of MN, according to the nerve B-mode dynamic imaging and the marker drawn at limb position 1 (Section 2.3). At each site, the MN thickness and Young’s modulus measurements were repeated three times. During the entire sweep, the operator placed the transducer covered with the coupling agent vertically and steadily on the skin and avoided applying pressure to the skin. Neither thickness nor Young’s modulus measurements included epineurium.

### 2.3 Limb positions

**FIGURE 2:**
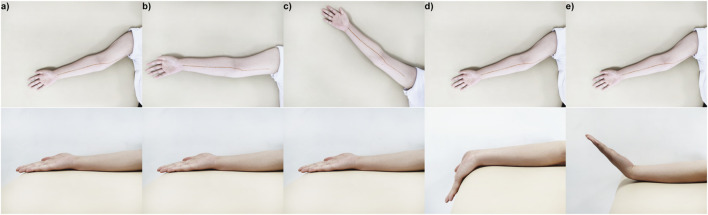
Five limb positions in the experiment: **(A)** limb position 1, the wrist was in a neutral position, the elbow extended at 180°, and the shoulder abducted at 60°; **(B)** limb position 2, the wrist was in a neutral position, the elbow extended at 180°, and the shoulder abducted at 90°; **(C)** limb position 3, the wrist was in a neutral position, the elbow extended at 180°, and the shoulder abducted at 120°; **(D)** limb position 4, the wrist extended at 45°, the elbow extended at 180°, and the shoulder abducted at 120°; **(E)** limb position 5, the wrist flexed at 45°, the elbow extended at 180°, and the shoulder abducted at 120°.

Each participant was examined in the supine position with five upper limb positions. The upper limb was initially posed in limb position 1 (Figure 2A), the wrist was in neutral position (Aseem, Williams et al., 2017), the elbow extended at 180°, and the shoulder abducted at 60°. In this position, the MN was relatively relaxed, which was appropriate for measuring the morphological and mechanical properties and used as a reference in Section 2.4 .

Then, two groups of shoulder and wrist rotation angles were designed to explore the influence of limb positions on the morphology and mechanical properties of MN, respectively. In the neutral wrist position and the 180° extension of the elbow, the shoulder was manipulated into 90° (limb position 2) (Figure 2B) or 120° (limb position 3) (Figure 2C); in the shoulder abduction at 60°, the elbow extension occurred at 180°, and the wrist extended at 45° (limb position 4) (Figure 2D) or flexed at 45° (limb position 5) (Figure 2E). In limb positions 4 and 5, the wrist was held in place passively by a brace. For each subject, the limb positions were within the maximum angular range that could be tolerated, and the test sequence was the same in order from limb position 1 to limb position 5. In addition, in the first scan of MN at limb position 1, the marker of the nerve was drawn on the arm surface with a harmless marker pen, using as a rough localization of MN in other limb positions.

### 2.4 Data analysis

All data analyses were performed on IBM Statistical Package for the Social Sciences (SPSS) and Origin 2018. The reproducibility of the data was calculated using ICC_3,1_ (Koo and Li, 2016), along with 95% confidence interval (95% CI).

The distribution curves of MN thickness and Young’s modulus in five limb positions were plotted on Origin 2018 using dotted line plots with upper and lower deviations showing the three measurements. At limb position 1, the differences among the wrist, mid-forearm, elbow, and mid-upper arm were examined. In addition, the discrepancies in the spatial distribution brought by shoulder (limb positions 1, 2, and 3) and wrist (limb positions 1, 4, and 5) movement groups were then explored separately. Differences between the data were tested separately using Friedman’s test and Bonferroni *post hoc* analysis (*p* < 0.05) in SPSS.

To further quantify the effects of shoulder and wrist rotations, four typical locations on the MN, namely, the wrist (wrist crease, 0–2 cm), mid-forearm (forearm midpoint, 8–10 cm), elbow (elbow, 16–18 cm), and mid-upper arm (upper arm midpoint, 24–26 cm), were selected and their differences in thickness and Young’s modulus were calculated, in detail, d_1-2_ = d_position1_ - d_position2_, E_1-2_ = E_position2_ - E_position1_, d_1-3_ = d_position1_ - d_position3_, E_1-3_ = E_position3_ - E_position1_, d_1-4_ = d_position1_ - d_position4_, E_1-4_ = E_position4_ - E_position1_, d_1-5_ = d_position1_ - d_position5_, E_1-5_ = E_position1_ - E_position5_. The variations were all designed to be positive to compare the magnitude. In Origin 2018, box plots were used to visualize the variation in the typical locations at the four limb positions.

## 3 Results

### 3.1 Repeatability analysis of MN thickness and Young’s modulus

The test–retest analysis results of the MN thickness and Young’s modulus in this study are summarized in Table 2. The results showed that the ICC_3,1_ of Young’s modulus was 0.996, with the 95% CI ranging from 0.979 to 0.998. The ICC_3,1_ of the thickness was 0.976, and the 95% CI was from 0.862 to 0.990. The data for both thickness and Young’s modulus have relatively good reliability.

**TABLE 2:** Retest reliability of median nerve thickness and Young’s modulus.

	*d*		E	
	ICC_3,1_	95% CI	ICC_3,1_	95% CI
Limb position 1	0.974	0.826–0.991	0.990	0.936–0.996
Limb position 2	0.975	0.821–0.991	0.987	0.931–0.995
Limb position 3	0.968	0.819–0.988	0.989	0.925–0.996
Limb position 4	0.971	0.805–0.990	0.995	0.969–0.998
Limb position 5	0.970	0.872–0.988	0.992	0.958–0.997
Total	0.976	0.862–0.990	0.996	0.979–0.998

### 3.2 Spatial distribution of MN thickness

**FIGURE 3:**
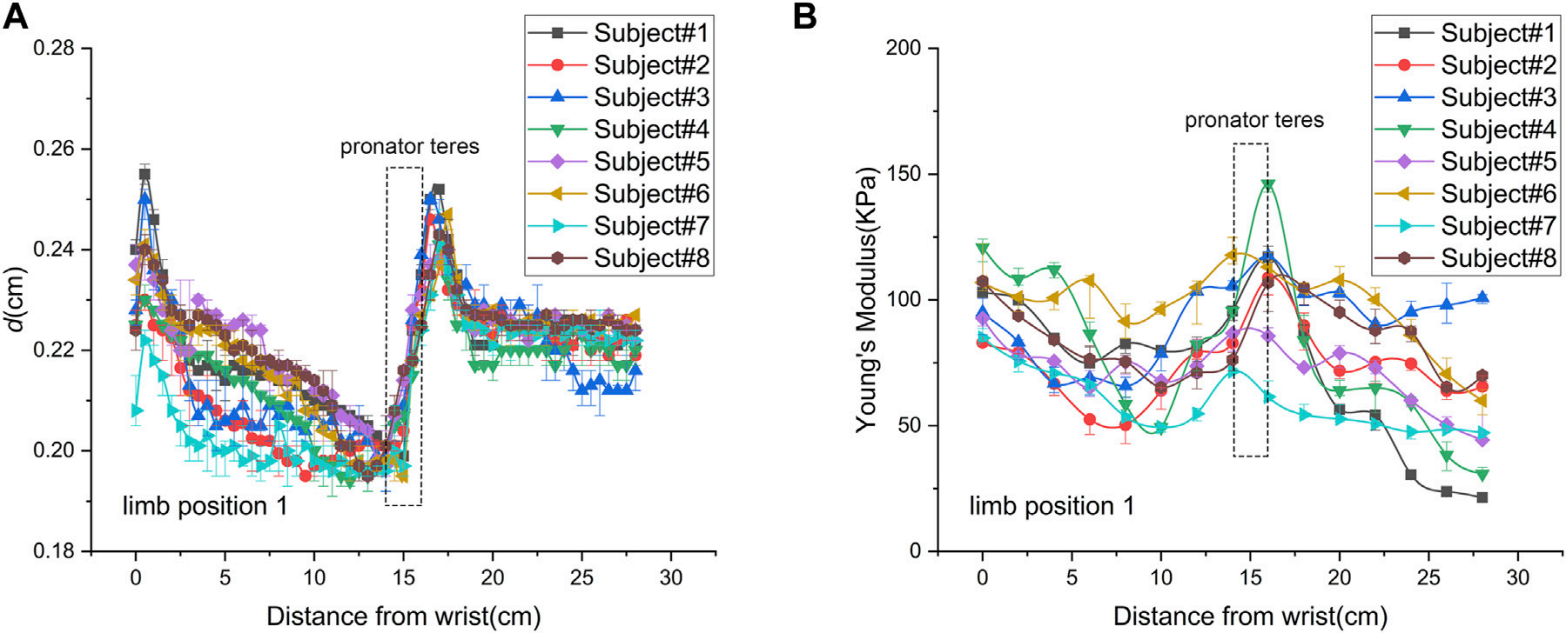
Spatial distributions of median nerve thickness and Young’s modulus of subject 8 at limb position 1: **(A)** thickness; **(B)** Young’s modulus.

The spatial distribution of MN thickness was characterized by the data measured at limb position 1 (Figure 3A). Nerve thickness increased and then decreased at the joints, declined slowly in the forearms, and was almost constant in the upper arms in proximal extension. As shown in Table 3, there were significant differences among the thickness values of the four typical locations (*p* < 0.0001). The MN is thickest at the elbow (0.236 ± 0.0073 mm) and is thinnest at the mid-forearm (0.208 ± 0.0065 mm). The thickness of MN at the wrist is a little thinner than that at the elbow.

**TABLE 3:** Median nerve thickness at four typical locations in five limb positions.

Limb position	Location	Mean ± SD (mm)
Limb position 1	Wrist^b^	0.230 ± 0.0097
	Forearm^d^	0.208 ± 0.0065
	Elbow^a^	0.236 ± 0.0073
	Upper arm^c^	0.223 ± 0.0036
Limb position 2	Wrist	0.225 ± 0.0072
	Forearm	0.209 ± 0.0074
	Elbow	0.237 ± 0.0082
	Upper arm	0.224 ± 0.0015
Limb position 3	Wrist	0.223 ± 0.0098
	Forearm	0.207 ± 0.0071
	Elbow	0.236 ± 0.0086
	Upper arm	0.225 ± 0.0029
Limb position 4	Wrist	0.214 ± 0.0078
	Forearm	0.203 ± 0.0032
	Elbow	0.235 ± 0.0062
	Upper arm	0.224 ± 0.0017
Limb position 5	Wrist	0.230 ± 0.0097
	Forearm	0.209 ± 0.0088
	Elbow	0.234 ± 0.0065
	upper arm	0.223 ± 0.0025

^a^ Elbow

^b^ Wrist

^c^ Upper arm

^d^ Forearm

### 3.3 The influence of limb positions on MN thickness

**FIGURE 4:**
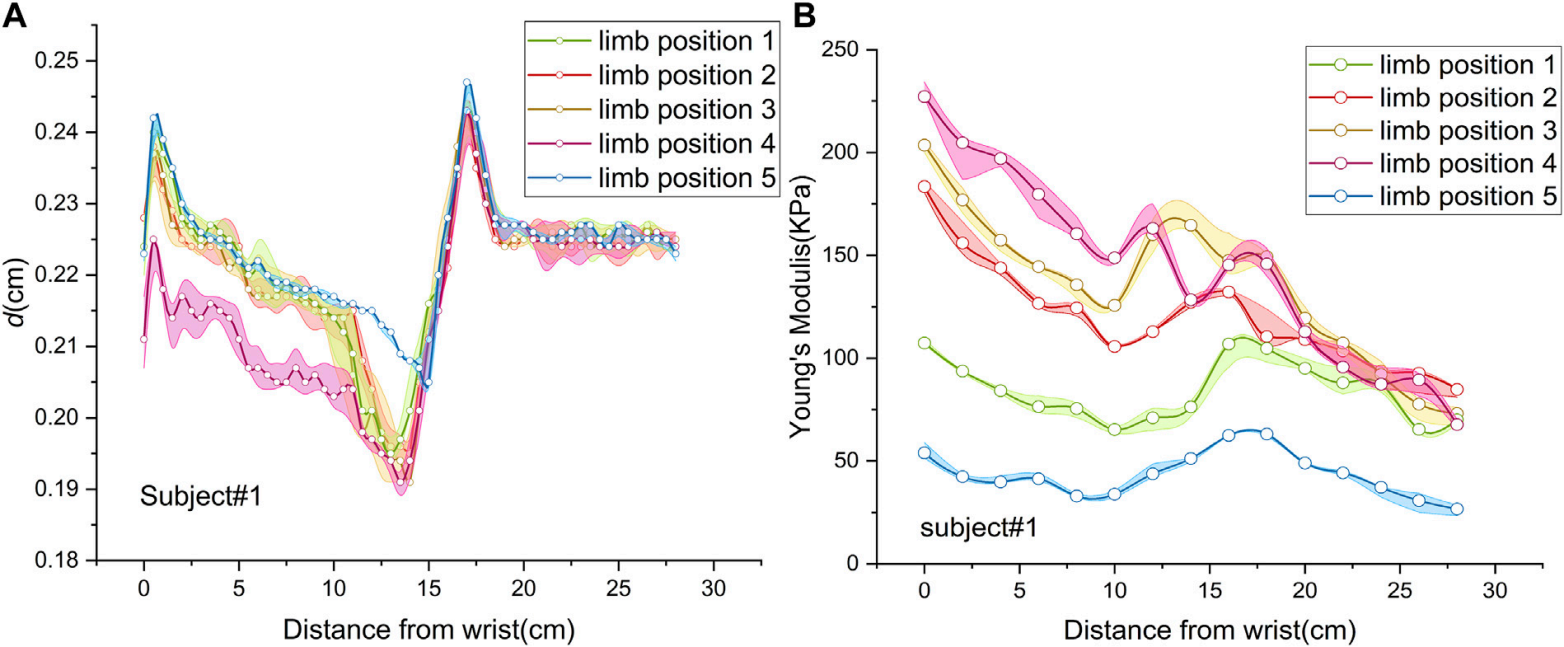
Spatial distributions of median nerve thickness and Young’s modulus of subject 1 at five limb positions: **(A)** thickness; **(B)** Young’s modulus.

Figure 4A shows the spatial distributions of MN thickness in five limb positions of subject 1. In the shoulder motion group, the spatial distributions were consistent without significant differences (*p* = 0.055). In the wrist motion group, the spatial distribution of thickness at limb position 4 significantly differed from that at limb position 1 (*p* < 0.001). However, there was no apparent difference between the thickness spatial distribution at limb position 5 and that at limb position 1 (*p* = 0.610). Only wrist extension produced visible changes in nerve thickness. Nevertheless, nerve thickness had the same distribution pattern in all five limb positions.

**FIGURE 5:**
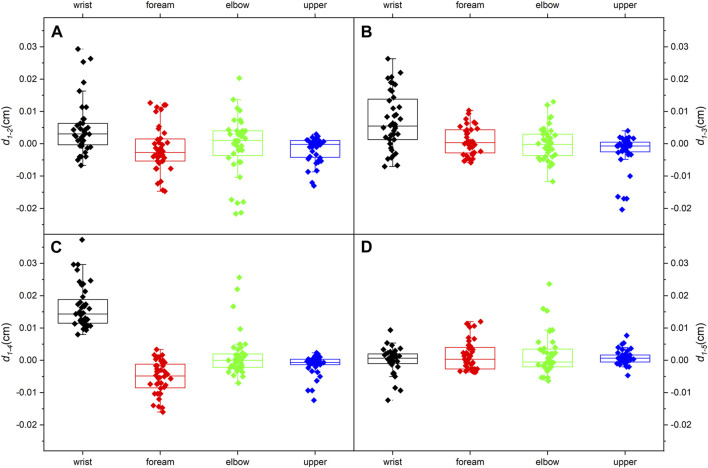
Median nerve thickness variations (95% range) at the four typical locations: **(A)** from limb position 1 to limb position 2; **(B)** from limb position 1 to limb position 3; **(C)** from limb position 1 to limb position 4; **(D)** from limb position 1 to limb position 5.

The thicknesses of the four locations at the five limb positions are shown in Table 3. For every 30° increase in the shoulder abduction angle, the variations in the thickness, d_1-2_ and d_1-3_, at the four locations were approximately 0 (Figures 5A, B). In the wrist motion group, the wrist extension caused a significant decrease in nerve thickness at the wrist (Figure 5C). Yet, the flexion of the wrist generated a faint effect on nerve thickness at the four locations (Figure 5D). Table 4 shows the 95% intervals of nerve thickness variations.

**TABLE 4:** Median nerve thickness variations (95% range).

	*d*_1-2_	*d*_1-3_	*d*_1-4_	*d*_1-5_
Wrist	0.0218,	0.00429,	0.0142,	−0.00128,
	0.00748	0.00983	0.0185	0.00125
Forearm	−0.00362,	−0.000424,	−0.00686,	0.0000204,
	0.000878	0.00232	−0.00364	0.00304
Elbow	−0.00340,	−0.00149,	−0.000666,	−0.000289,
	−0.00243	0.00177	0.00358	0.00369
Upper arm	−0.00292,	−0.00423,	−0.00235,	0.000177,
	−0.000454	−0.000614	−0.000343	0.00161

### 3.4 Spatial distribution of MN Young’s modulus

The spatial distribution of MN Young’s modulus used the data from limb position 1 (Figure 3B). Most parts of Young’s modulus distribution are similar with that of thickness, except the wrist and the pronator teres. In proximal extension, the nerve stiffness decreased at the wrist and increased through the pronator teres. Table 5 shows that Young’s modulus of the nerve was significantly higher at the elbow and wrist than that at the forearm and upper arm midpoints (*p* < 0.001). There was neither a discernible difference in nerve stiffness between the elbow and wrist nor between the mid-forearm and the mid-upper arm, which is different from the results of thickness.

**TABLE 5:** Median nerve Young’s modulus at four typical locations in five limb positions.

Limb position	Location	Mean ± SD (KPa)
Limb position 1	Wrist^a^	94.60 ± 13.54
	Forearm^b^	68.33 ± 14.84
	Elbow^a^	97.11 ± 22.40
	Upper arm^b^	62.62 ± 22.66
Limb position 2	Wrist	153.84 ± 26.10
	Forearm	112.05 ± 23.65
	Elbow	125.59 ± 24.30
	Upper arm	92.55 ± 23.11
Limb position 3	Wrist	188.93 ± 35.25
	Forearm	129.43 ± 16.15
	Elbow	148.92 ± 23.89
	Upper arm	100.99 ± 20.69
Limb position 4	Wrist	224.65 ± 49.72
	Forearm	143.87 ± 44.98
	Elbow	122.19 ± 42.09
	Upper arm	80.20 ± 29.46
Limb position 5	Wrist	63.44 ± 22.72
	Forearm	39.07 ± 12.97
	Elbow	67.55 ± 16.14
	Upper arm	42.27 ± 15.85

^a^ ^Elbow^

^b^ ^Wrist^

### 3.5 The influence of limb positions on MN Young’s modulus

**FIGURE 6:**
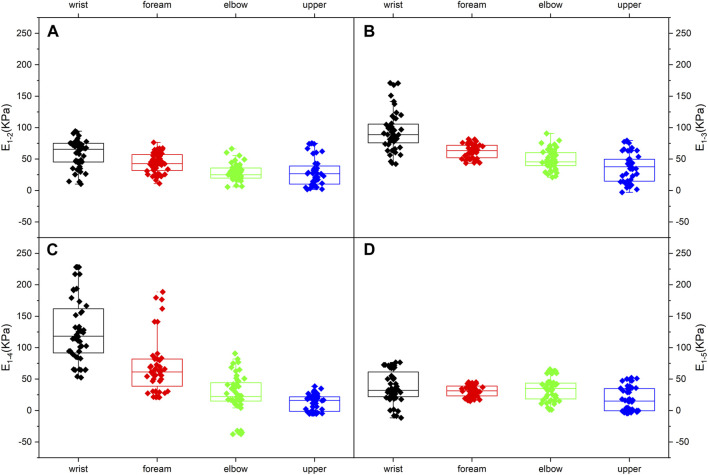
Median nerve Young’s modulus variations (95% range) at the four typical locations: **(A)** from limb position 1 to limb position 2; **(B)** from limb position 1 to limb position 3; **(C)** from limb position 1 to limb position 4; **(D)** from limb position 1 to limb position 5.

Figure 4B depicts MN Young’s modulus distributions of subject 1 at five limb positions. Both shoulder and wrist motions affected nerve stiffness apparently. Young’s modulus measured at limb positions 2 and 3 differed substantially from those measured at limb position 1 (*p* < 0.001). Furthermore, the differences in Young’s modulus among limb positions 1, 4, and 5 were all statistically significant (*p* < 0.001). Table 5 lists the MN’s Young’s modulus of four locations at five limb positions. In the shoulder motion group, Young’s modulus increased continuously with sequential abduction of the shoulder (Figures 6A, B). In addition, there was a marked increase in nerve stiffness at limb position 5 (Figure 6C). Limb position 5 resulted in a statistically significant decrease in nerve stiffness (Figure 6D). Overall, the stiffness variations in the four locations were non-uniform. The Young’s modulus variation in the nerve is maximum at the wrist and is minimum at the mid-upper arm (Table 6).

**TABLE 6:** Median nerve Young’s modulus variations (95% range).

	E_1-2_	E_1-3_	E_1-4_	E_1-5_
Wrist	52.876, 65.599	85.321, 103.332	112.729, 142.908	30.778, 45.251
Forearm	39.072, 48.373	59.571, 65.724	56.285, 80.894	28.517, 33.916
Elbow	24.661, 32.297	44.433, 53.457	16.568, 34.748	29.088, 39.578
Upper arm	22.934, 35.986	30.368, 43.595	9.290, 16.646	12.121, 23.037

## 4 Discussion

Previous studies have shown that the CSA, anteroposterior diameter (AP diameter), and transverse diameter of the nerve are important parameters for quantitatively describing the morphology (Kuo, Leong et al., 2001). The CSA, excellent in neuropathy diagnosis, failed to show a significant correlation with Young’s modulus (Rugel, Franz et al., 2020; Tang, Zhu et al., 2021). Young’s modulus calculated along the long-axis direction approximates the stiffness of the nerve, while the CSA is measured in the cross section. Therefore, the AP diameter of the nerve was applied in this study. In addition, CTS occurs more often in female individuals (Buchberger, Schon et al., 1991). The MN in female individuals was thought to be more sensitive to limb motions, so young female individuals were chosen as subjects.

### 4.1 Spatial distribution of MN thickness

In Figure 3A, MN thickness exhibits consistency in all subjects. The distribution pattern shows the folded shape. The folded points are at the carpal tunnel entrance, proximal pronator teres, and elbow. There is a noticeable decrease in the AP diameter near the carpal tunnel entrance, presumably due to the flattening of the MN. The increased flattening facilitates the adaptation to the carpal tunnel and contributes to the dispersion of the stress on the nerve. Overall, the MN thickness of the forearm slowly reduces as it extends proximally, while the upper arm nerve is thicker and invariant. The MN thins as it penetrates the pronator teres and obviously thickened by the elbow. Variations in CSA, branching, and location are presumed to account for these changes. As the nerve is closer to the skin, the nerve is thicker, while as the nerve goes deeper, the nerve becomes thinner, which may be related to the flattening of the MN.

### 4.2 The influence of limb positions on MN thickness

We assumed that an increase in the shoulder extension angle would subject the nerve to more tremendous tensile strength and result in an overall thinning (Topp and Boyd, 2006). However, no detectable changes in thickness occur at any of the four typical locations, and the overall thickness distribution is identical in the shoulder motion group. It considers that the shoulder motion does not bring visible changes to the MN thickness.

The extension of the carpal joint causes significant thinning in the wrist, and the thickness distribution is quite different. The consequence is compatible with the previous study (Keir and Rempel, 2005). The wrist extension generates stronger tensile properties and exposes the nerve to compression from the carpal tunnel. As a result, the MN thickness at the wrist becomes distinctly lower. However, the flexion of the wrist lacks effect on thickness. This result is inconsistent with the literature (Loh, Yeoh et al., 2019), which may be related to the subject population (BMI, gender, age, etc.). The loading on the MN in the carpal tunnel during wrist flexion has not been reported. Therefore, the change in the MN thickness in the wrist flexion is difficult to speculate.

### 4.3 Spatial distribution of MN Young’s modulus

According to Figure 3, the Young’s modulus spatial distributions are also in a folded shape. The folded points are at distal pronator teres and elbow, which are different from those of thickness distribution. These differences occur to the locations where the nerve is thinner and harder.

Young’s modulus in the forearm generally decreased proximally. The nerve stiffness increased as the MN passed through the pronator teres, with the upward trend continuing up to the supracondylar humerus, and steadily declined in the upper arm. MN Young’s modulus under the pronator teres is largest throughout the non-articular region. This is because the MN location is close to the joint and covered with the muscle.

There is a significant difference in nerve stiffness between the joints and the non-joint locations. Previous studies show apparent structural differences between articular and non-articular nerves (Phillips, Smit et al., 2004; Mason and Phillips, 2011). At limb position 1, the elbow and wrist are equally extended at 180°, and the nerve at the joint is under tension due to the lengthening of the nerve bed (Phillips, Smit et al., 2004). The tension gets weaker as the nerve is further away from the joint, and the nerve stiffness decreases. Moreover, the MN at the joint is closer to the bone surface, leading to a higher Young’s modulus (Bortolotto, Turpini et al., 2017).

### 4.4 The influence of limb positions on MN Young’s modulus

For the shoulder motion group, Young’s modulus increases in response to the shoulder abduction angle increments. The distributions differ among the three limb positions because of the higher nerve tension (Greening and Dilley, 2017; Rugel, Franz et al., 2020) caused by the increasing abduction angle. Higher nerve tension leads to higher stiffness. MN Young’s modulus shows non-linear increases. The numerical relationship of increments is E_1-2_<E_1-3_<2E_1-2_ (Figures 6A, B). This non-linear relationship is attributed to the higher stiffness and lower elasticity of MN at limb position 2. The nerve deformation is limited, and Young’s modulus increment is consequently less. In other words, the ability of the nerve to adapt to limb motion is constrained.

Furthermore, the increments of Young’s modulus of the four locations do not appear to decrease distally but show the opposite trend. It is speculated that the test range does not extend to the axilla due to the insufficient space of the probe. In addition, the operator might not place the probe in the optimal position when measuring Young’s modulus in the upper arm.

The effect of carpal extension on the MN stiffness is the most intense. The Young’s modulus at the wrist is much higher than at other locations, which may explain the high incidence of CTS to some extent. The above section has discussed the reasons for this variation.

Wrist flexion produces an overall decrease in neural stiffness. In addition, the variation in Young’s modulus at typical locations, E_1-4_ and E_1-5_, is essentially the same, except for the wrist. This phenomenon suggests that the changes in MN Young’s modulus, except for the wrist, are primarily derived from nerve tension. The distribution of tension induced by wrist motions can be obtained from the variations in Young’s modulus (Figures 6C, D). The closer the wrist, the higher the Young’s modulus variation and the greater the nerve tension induced by wrist extension.

The nerve tension is caused by the elongation of the nerve bed during the joint movements. Elongating the nerve increases its strain, and the longitudinal tensile load straightened the wavy connective tissue and axons in the endoneurial compartment (Sunderland, 1990; Kwan, Wall et al., 1992; Pourmand, Ochs et al., 1994). Moreover, elongation of the nerve is likely to cause a reduction in the cross-sectional area, called transverse contraction (Millesi, Zöch et al., 1995). The transverse contraction will increase the pressure in the endoneurial compartment. A theoretical model suggested that the biomechanical properties of the nerve under tension are highly related to the outer connective tissue tube or sheath constraining the inner pressurized neural core (Millesi, Zöch et al., 1995; Walbeehm, Afoke et al., 2004). To resist the transverse contraction, the neural core generated more pressure, which leads to increased nerve stiffness (Millesi, Zöch et al., 1995). When the tension is removed, it is likely that a combination of elasticity of the connective tissues and pressure within the neural core will allow recoiling of the nerve to nearly the original CSA and length (Topp and Boyd, 2006).

At the wrist, the loading of the MN is more complex, which is why we discussed the morphological and mechanical properties at limb position 1. Wrist extension leads to nerve stretching with raised tension and narrows the carpal tunnel with increasing compression (Loh, Yeoh et al., 2019). Although wrist flexion releases nerve tension, the nerve is subject to strike from the underlying tendons. In contrast, the Young’s modulus at the wrist is statistically decreased in this study. We suggest that the upward arching of the tendons during wrist flexion expands the carpal tunnel outward as well. Therefore, although the below tendons will hit the MN, the pressure from above will be reduced, and the general stiffness will diminish.

### 4.5 Locations

Figures 5, 6 show that the MN at the wrist is the most sensitive and distinct to limb movements, which could also explain that CTS is one of the most prevalent compressive neuropathies. Changes in biomechanics and morphology are often associated with changes in the microstructure. Nerves will develop pathology if they are in an uncomfortable position for a long period (Lee, Yang et al., 2012). Unlike thickness, Young’s modulus is more sensible and prominent to limb motions. Thus, Young’s modulus can provide a precise assessment of the MN and has potential for early diagnosis.

The study explored the morphological and biomechanical characteristics of the MN with high-frequency ultrasound and SWE. Then, the effects of continuous 30° of shoulder extension and wrist extension, flexion of 45° on the overall trend, and local quantitative changes were explored. There are several shortcomings in this study: 1) the recruited subject population was only healthy young women without taking gender and age into account. These findings can provide theoretical study foundation while not being extended to all clinical groups. 2) Moreover, research lacks the studies related to the pathology and pathologic tracing of the MN. SWE is a useful tool to visualize nerve biomechanics. With this technique, we can study the changes in neuro-biomechanics introduced by compression neuropathy and the nerve regeneration process after surgery. This information could be helpful for early diagnosis and prognosis of the MN compressive lesions. 3) At 60° of shoulder extension, since the space was not enough to place the probe close to the axilla, the trial tested of the MN range was not adequate. In the future, further studies will be performed to break the above limitations and optimize the conclusions.

## Data Availability

The raw data supporting the conclusion of this article will be made available by the authors, without undue reservation.
