# Supplementary material for: Non-invasive in vivo study of morphology and mechanical properties of the median nerve
Source: Front Bioeng Biotechnol. 2024 Apr 11;12:1329960. doi: 10.3389/fbioe.2024.1329960 (PMC11043530; doi:10.3389/fbioe.2024.1329960)
Supplement: Supplementary file 1 [file DataSheet1.DOCX]

Supplementary Material

Non-invasive in vivo study of morphology and mechanical properties of the median nerve

Ruixia Xu*, Lei Ren, Jing Liu

*** Correspondence:** Lei Ren: [lren@jlu.edu.cn](mailto:lren@jlu.edu.cn) Jing Liu: [jingliu@jlu.edu.cn](mailto:jingliu@jlu.edu.cn)

# Supplementary Figures







**Supplementary Figure 1.** The spatial distributions of median nerve thickness and Young’ s modulus of subject 2 at five limb positions a) thickness; b) Young’s modulus.







**Supplementary Figure 2.** The spatial distributions of median nerve thickness and Young’ s modulus of subject 3 at five limb positions a) thickness; b) Young’s modulus.







**Supplementary Figure 3.** The spatial distributions of median nerve thickness and Young’ s modulus of subject 4 at five limb positions a) thickness; b) Young’s modulus.







**Supplementary Figure 4.** The spatial distributions of median nerve thickness and Young’ s modulus of subject 5 at five limb positions a) thickness; b) Young’s modulus.







**Supplementary Figure 5.** The spatial distributions of median nerve thickness and Young’ s modulus of subject 6 at five limb positions a) thickness; b) Young’s modulus.







**Supplementary Figure 6.** The spatial distributions of median nerve thickness and Young’ s modulus of subject 7 at five limb positions a) thickness; b) Young’s modulus.








**Supplementary Figure 7.** The spatial distributions of median nerve thickness and Young’ s modulus of subject 8 at five limb positions a) thickness; b) Young’s modulus.
